# Ferroptosis: the balance between death and survival in colorectal cancer

**DOI:** 10.7150/ijbs.96828

**Published:** 2024-07-02

**Authors:** Shiying Fan, Lujia Zhou, Wenjie Zhang, Daorong Wang, Dong Tang

**Affiliations:** 1 Clinical Medical College, Yangzhou University, Yangzhou, 225000, P. R. China.; 2 School of Medicine, Chongqing University, Chongqing, 400030, P. R. China.; 3 Department of General Surgery, Institute of General Surgery, Northern Jiangsu People's Hospital Affiliated to Yangzhou University, Yangzhou, 225000, P. R. China.

**Keywords:** ferroptosis, colorectal cancer, lipid peroxidation, anticancer treatment

## Abstract

Colorectal cancer (CRC) is a common malignant tumor associated with high morbidity and mortality. Despite an increase in early screening and treatment options, people with CRC still have a poor prognosis and a low 5-year survival rate. Therefore, mining more therapeutic targets and developing means of early diagnosis and determining prognosis are now imperative in the clinical treatment of CRC. Ferroptosis is a recently identified type of regulated cell death (RCD) characterized, which is identified by the accumulation of iron-dependent lipid peroxidation, thereby causing membrane damage and cell death. Recent studies have shown that ferroptosis is associated with tumors, including CRC, and can be involved in CRC progression; however, the underlying mechanisms are complex and heterogeneous and have not been thoroughly summarized. Therefore, this study reviewed the roles of ferroptosis in CRC progression to target ferroptosis-related factors for CRC treatment. The significance of ferroptosis-related biomarkers and genes in the early diagnosis and prognosis of CRC was also investigated. Furthermore, the limitations of ferroptosis studies in the current treatment of CRC, as well as future research perspectives, are discussed.

## Introduction

Colorectal cancer (CRC), a malignant tumor of the digestive system, is marked by the uncontrolled proliferation and survival of aberrant cells in the colon or rectum. As the disease progresses to an advanced stage, the tumor foci can migrate into other normal tissues or sites, which is associated with high morbidity and mortality 1, 2. In 2020, CRC accounted for 10% of new cancer cases worldwide 3. The 5-year relative survival of patients with CRC in America is 91% for localized disease and 14% for distant disease 4. Currently, the primary treatments for CRC are surgical resection, radiotherapy, chemotherapy, immunotherapy, and targeted therapy 5. However, the therapeutic targets for CRC are limited owing to the many unidentified intermediate molecules involved in CRC pathogenesis, which hinders the clinical effectiveness of treatments 6. Therefore, exploring the key molecules involved in CRC progression as potential therapeutic targets is crucial to increase the survival rate. In addition, early diagnosis can significantly increase the survival rate of patients with CRC 7. However, owing to the complex biological characteristics of CRC and a shortage of highly sensitive and specific biomarkers, early screening for CRC still relies on invasive examinations such as endoscopy 7. Consequently, identifying new biomarkers is critical for developing novel approaches to the early, non-invasive diagnosis and prognosis of CRC.

Apoptosis can be induced in CRC cells by elevating reactive oxygen species (ROS) levels, lowering antioxidant glutathione (GSH) levels, or deactivating glutathione peroxidase 4 (GPX4), all of which are also central to or associated with ferroptosis 8-10. The concept of ferroptosis was first proposed by Dixon in 2012, and its essence is the impaired metabolism of intracellular lipid oxides, which in turn is abnormally metabolized under the catalysis of iron ions, generating large amounts of lipids, disrupting intracellular redox homeostasis, attacking biomolecules, and triggering cell death, which is a type of iron-ion-dependent non-apoptotic cell necrosis 11, 12. Activating ferroptosis aids in CRC treatment, whereas inhibiting ferroptosis may induce CRC development or the emergence of drug resistance 9, 13. As a result, manipulating ferroptosis could be useful in CRC treatment. Recognizing the significance of early diagnosis and accurate prognosis in improving the efficacy of CRC treatment, scientists have identified ferroptosis-related proteins and genes as potential biomarkers for the diagnosis and prognosis of CRC 14, 15. This review focused on the role and mechanism of ferroptosis in CRC development. Additionally, the significance of targeting ferroptosis core regulators in CRC treatment and the role of ferroptosis-related molecules in early diagnosis and prognosis monitoring were discussed.

## Overview of ferroptosis

### Core concepts and the three elements of ferroptosis

In the presence of iron, ROS in cells converts polyunsaturated fatty acids (PUFAs) on oxidized lipid membranes into lipid peroxides, causing membrane damage and cell death 16. This process is termed ferroptosis, a newly discovered regulated cell death (RCD) 16. RCD is a death mode that occurs in a physiological state or upon failing to adapt to stress, and is under active and orderly control by the cells 17. Currently, the identified RCD modes include autophagy, apoptosis, necroptosis, pyroptosis, and ferroptosis 18. Various morphological, biochemical, immunological, and genetic characteristics set it apart from other types of RCD 19.

The primary cause of ferroptosis-mediated cell death is the imbalance between intracellular lipid ROS generation and degradation 16. The reduced antioxidant capability of cells causes excess iron to trigger ferroptosis by producing deadly ROS through the Fenton reaction 20. ROS can carry extremely unstable energy and are prone to uncontrolled energy loss leading to cell death, and this is the root cause of the harmful effects of ROS on organisms 21. Iron ions (Fe^2+^/Fe^3+^), which are major inducers of ferroptosis, can contribute to the formation of ROS through enzymatic or non-enzymatic reactions 22. Iron from plasma typically enters cells as Fe^3+^ through transferrin and its receptor, where it is reduced to Fe^2+^ by ferric reductase 23. Fe^2+^ catalyzes the Fenton reaction, an important cause of rapid and dramatic ferroptosis, to break the peroxide bond from H_2_O_2_ and produces the highly oxidized hydroxyl radical, which is the most potent oxidant in ROS 24, 25. Subsequently, ROS or lipoxygenase (LOX) oxidizes the nontoxic phospholipids containing PUFAs (PL-PUFA) to the toxic peroxidized lipid, PL-PUFA-OOH 26. PUFAs, the most easily peroxidized lipids among the cell membrane components, incorporated into the membrane to form the PUFA-containing phospholipid, PL-PUFA 26. PL-PUFA-OOH converted from PL-PUFA is the lipid peroxide in ferroptosis 27. Eventually, the accumulation of PL-PUFA-OOH damages the cell membrane, leading to ferroptosis 27. This is the basic process of ferroptosis.

**Figure 1:**
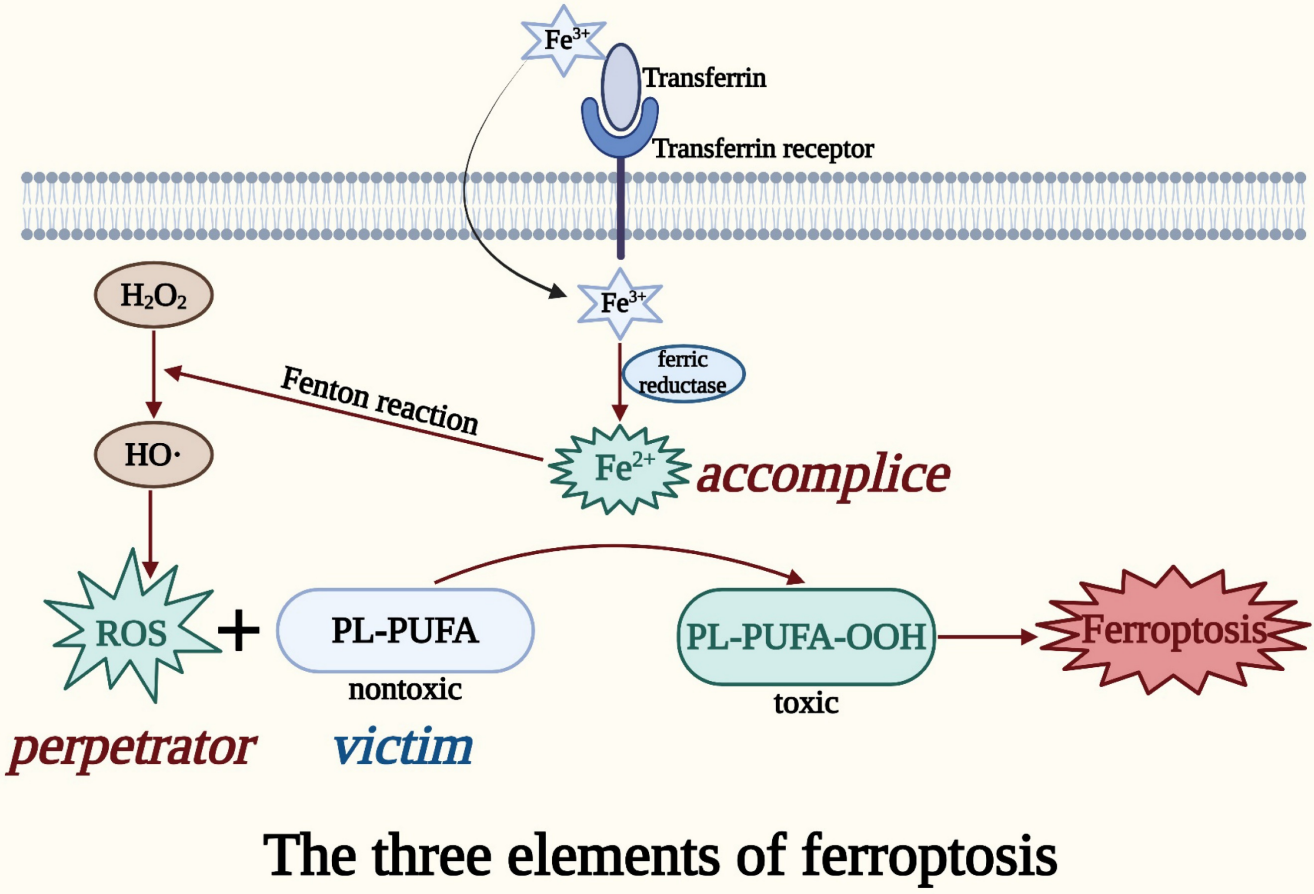
** The three elements of ferroptosis.** ROS in cells produces lipid peroxides from PUFAs on oxidized lipid membranes in the presence of iron, thereby causing membrane damage and cell death, which is called ferroptosis. The perpetrator ROS, the accomplice iron, and the victim PL-PUFA can be regarded as the three elements of ferroptosis.

Taken together, the perpetrator ROS, the accomplice iron, and the victim PL-PUFA might be considered the three elements of ferroptosis (**Figure 1**). The accumulation of the three elements of ferroptosis in an organism can be used as susceptibility factors for the occurrence of ferroptosis, which is important for the treatment and diagnosis of CRC 24.

## Primary defense pathways in ferroptosis

**Figure 2:**
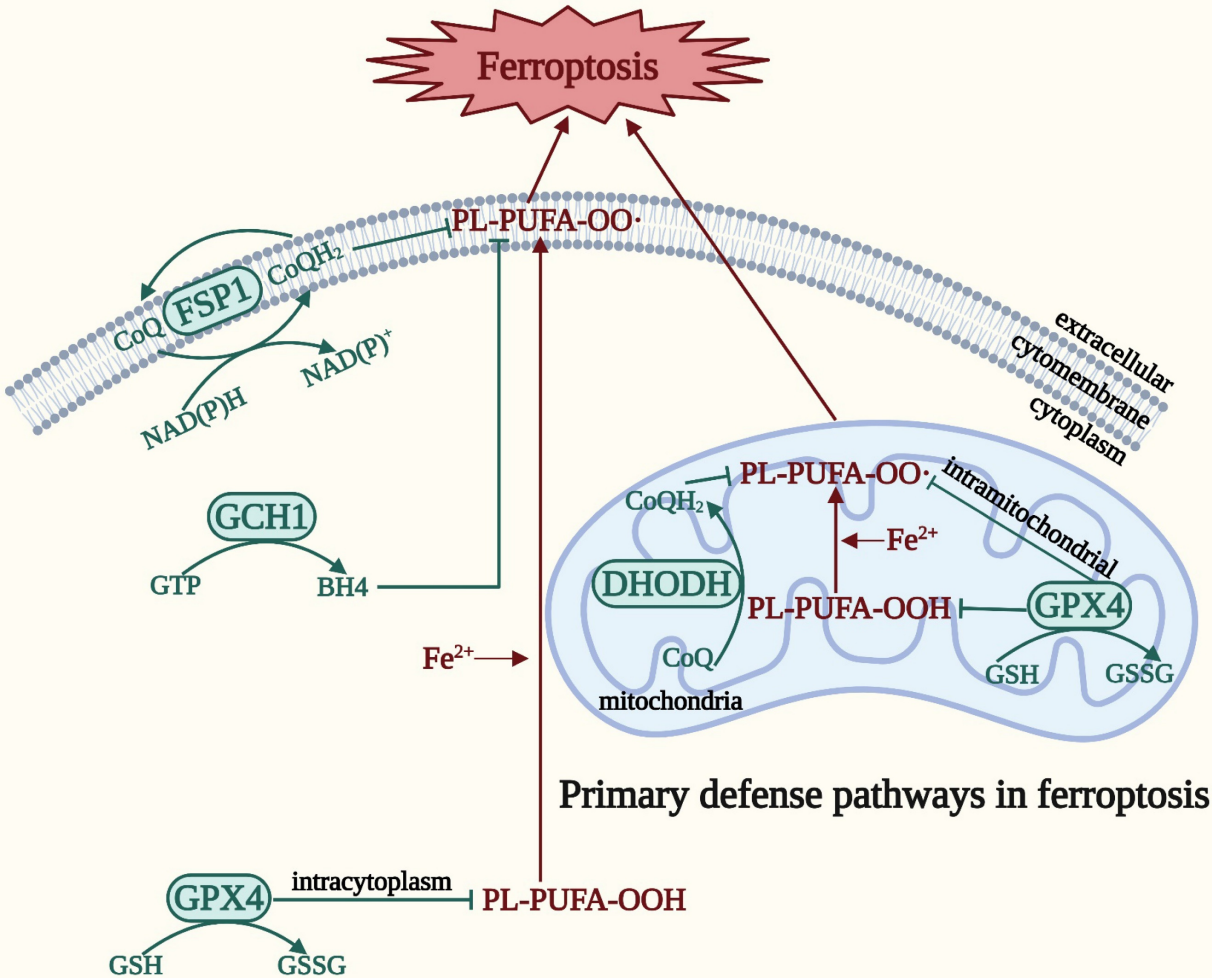
** Four main defense pathways in ferroptosis.** The GPX4-GSH signaling pathway is primarily located in the cytoplasm and mitochondria. The DHODH-CoQ signaling pathway is primarily located in the mitochondria. The FSP1-CoQ signaling pathway and the GCH1-BH4 signaling pathway are primarily located in the cytoplasm.

In order to survive against ferroptosis, the body has developed a defense system. There are four main defense pathways in ferroptosis: the GPX4-GSH, ferroptosis suppressor protein 1 (FSP1)-coenzyme Q (CoQ), dihydroorotate dehydrogenase (DHODH)-CoQ, and guanosine 5′-triphosphate cyclohydrolase-1 (GCH1)-tetrahydrobiopterin (BH4) signaling pathways 10 (**Figure 2**). In contrast to the relatively static, slow process in ferroptosis represented by the three elements, the defense pathway is a relatively dynamic expression of the process of ferroptosis, and is the remedy that prevents the cell from moving toward eventual death after ferroptosis has occurred 10. GPX4 is a selenoprotein that can break down both relatively complex lipid peroxides and small molecule peroxides, and can also convert cytotoxic lipid hydroperoxides into nontoxic lipid alcohols, preventing the generation and accumulation of deadly ROS in order to protect the integrity of the membrane 28, 29. GPX4 employs GSH, a tripeptide antioxidant comprising glutamate, cysteine, and glycine, as a cofactor to degrade hydroperoxide 28, 30. An indirect method of inactivating GPX4 is GSH depletion, which further lowers cellular antioxidant capability and enhances the buildup of lipid ROS and the consequent ferroptosis 30. Thus, ferroptosis can be induced by impeding GSH synthesis and absorption or hastening its breakdown. In addition, CoQ10 is an endogenous antioxidant that protects cells from ferroptosis by blocking the propagation of lipid peroxides 31. FSP1 has been classified as a new GSH-independent ferroptosis suppressor that catalyzes the regeneration of CoQ10, thereby inhibiting lipid peroxidation 32. Therefore, the FSP1-CoQ signaling pathway exists as an independent parallel system that synergistically inhibits phospholipid peroxidation and ferroptosis with the GPX4-GSH signaling pathway 32. The DHODH-CoQ signaling pathway blocks mitochondrial lipid peroxidation and thus ferroptosis 33. DHODH, which is located on the outer surface of the inner mitochondrial membrane and operates in parallel with mitochondrial GPX4 (but independently of cytoplasmic GPX4 or FSP1), inhibits ferroptosis in the inner mitochondrial membrane by reducing CoQ to panthenol, a free-radical trapping antioxidant with anti-ferroptosis activity 33. The GCH1-BH4 signaling pathway is the primary GPX4 non-dependent ferroptosis regulatory system 34. BH4 biosynthesis requires GCH1 catalysis, thus inducing lipid remodeling and inhibits ferroptosis by selectively preventing depletion of phospholipids with two polyunsaturated fatty acyl tails 34, 35. The degree of cellular resistance to ferroptosis is substantially determined by the expression level of GCH1. Reduced BH4 resulting from genetic or pharmacological suppression of GCH1 can increase lipid peroxidation and ferroptosis 34. Conversely, overexpression of GCH1 selectively increases BH4 biosynthesis and reduces ROS production 36. Moreover, BH4 can convert phenylalanine to tyrosine, which can subsequently be converted into 4-OH-benzoate, a precursor of CoQ10, facilitating the production of CoQ10 to inhibit ferroptosis 35. Accordingly, these processes coordinate and precisely control ferroptosis by linking the GCH1-BH4 signaling pathway to the FSP1-CoQ signaling pathway.

Taken together, these four ferroptosis defense pathways are interconnected yet regulate ferroptosis relatively independently, making the ferroptosis defense network increasingly complete. However, unidentified signaling molecules in these pathways need to be discovered, and other ferroptosis inhibitory pathways still require investigation.

## Ferroptosis in CRC

### Factors that increase CRC cell susceptibility to ferroptosis

Recently, ferroptosis has attracted much attention in the cancer research community, in part because it is a unique form of cell death with its own mechanism and morphology 37. The close relationship between ferroptosis and CRC has been confirmed by the presence of multiple ferroptosis-inducing factors in CRC cells 38. The level of ROS, the perpetrator in ferroptosis, is usually higher in CRC cells than in normal counterparts, and hence CRC cells are more susceptible to ferroptosis 39, 40. In addition, as the necessary component for the transfer of the accomplice iron in the ferroptosis three elements, transferrin receptor 1 (TfR1) is also overexpressed in CRC tissues, which is a type II transmembrane glycoprotein commonly expressed on the cell surface 41. It is a crucial protein involved in controlling iron intake and cell growth, as well as a major regulator of cellular iron homeostasis 41. Therefore, CRC cells containing excess iron and TfR1 are theoretically more prone to ferroptosis. Moreover, CRC cells also contain ferroptosis-inducing factors that promote the transformation of the victim in ferroptosis. For example, CRC cells expressed high levels of high levels of LOX, which can oxidize the victim PL-PUFA to the toxic lipid peroxide PL-PUFA-OOH 42. LOX is thought to be a central player in ferroptosis, as pharmacological inhibition of LOX has been observed to be cytoprotective, so high levels of LOX should make CRC cells more susceptible to ferroptosis 43.

However, CRC cells overexpress the aforementioned ferroptosis susceptibility factors, certainly, not for self-attack, but because these factors can be beneficial for their proliferation or invasion in the first place. Although ROS is regarded as the perpetrator in ferroptosis, higher-than-normal levels of ROS in CRC can lead to cellular damage, DNA mutations, and inflammation, which can promote the proliferation and migration of CRC cells 39, 44. Additionally, CRC cells proliferation also requires large amounts of iron 45. The increased need for iron uptake leads to high TfR1 expression, and hence higher levels of TfR1 are primarily for survival rather than for ferroptosis 46. Furthermore, while high levels of LOX make CRC cells susceptible to ferroptosis, there is evidence that blocking LOX inhibits CRC progression 42, 43. LOX inhibitors enhanced phosphatase and tensin homolog deleted on chromosome 10 (PTEN) activity to inhibit the phosphatidylinositol 3-kinase (PI3K)/ protein kinase B (AKT) pathway, thus promoting cell survival and inhibiting apoptosis, which slows CRC progression 42. This suggests that high levels of LOX contribute to the development of CRC. These studies indicated that ferroptosis susceptibility factors can be viewed as a double-edged sword in CRC survival. It is possible that the level of expression of these ferroptosis susceptibility factors causes the different outcomes of CRC cell survival or death: the level of these factors expressed in CRC cells may not be sufficient to cause ferroptosis yet, but can facilitate the growth of CRC tissues. However, the above speculations have not yet been confirmed experimentally, which may be a future research direction with far-reaching implications for targeting ferroptosis for the treatment of CRC.

### Factors influencing CRC cell to ferroptosis

However, CRC cells do not undergo ferroptosis as assumed, not only because of the double-edged-sword-like susceptibility factor, but also largely because the defense pathway for ferroptosis is unusually active in CRC cells, which prevents CRC cells from undergoing ferroptosis 47, 48. For instance, GPX4, the key factor in the GPX4-GSH signaling pathway, is highly expressed in CRC tissues and high GPX4 expression is strongly associated with a poor prognosis in CRC 47. The active ferroptosis defense system prevents ferroptosis and ultimately leads to CRC progression.

In addition, potential negative regulators of ferroptosis are expressed in CRC cells. The expression of the TP53-induced glycolysis and apoptosis regulator (TIGAR) is significantly higher in CRC tissues than in neighboring normal tissues 49. Erastin-induced ferroptosis in CRC cells was significantly increased upon the knockdown of TIGAR, indicating that low TIGAR levels make CRC cells more susceptible to erastin-induced ferroptosis and that TIGAR may function as a ferroptosis inhibitor during CRC development 50. Low levels of TIGAR increased the production of lipid peroxidation and promoted the accumulation of lipid peroxidation product malondialdehyde (MDA), but no significant change was observed in iron levels, suggesting that TIGAR is a potential target for ferroptosis-based CRC treatment by regulating ROS 50. Similarly, cytochrome P450 1B1 (CYP1B1) is overexpressed in CRC, and CYP1B1 promotes CRC cells resistance to ferroptosis via alleviating lipid peroxidation, resulting in a poor prognosis in patients with CRC 51. These highly-expressed negative regulators of ferroptosis facilitate CRC development.

To summarize, the presence of ferroptosis-resistant factors in CRC cells, including the abnormally active ferroptosis defense signaling pathway and the presence of ferroptosis-negative regulatory molecules, allows CRC cells to evade ferroptosis to continue proliferating. Therefore, targeting ferroptosis-resistant factors has the potential to broaden the pathway for the treatment of CRC. Existing research has shown the essential function of ferroptosis in the development of CRC, with a view to providing more emerging targets for the clinical treatment of CRC, but more experiments are still needed to explore specific mechanisms.

## Target ferroptosis for the treatment of CRC

CRC is still one of the diseases that pose the greatest risk to human health 52. Patients with CRC typically experience rectal bleeding and abdominal pain, which have a significant impact on their quality of life 53. CRC is the second leading cause of cancer death in both men and women, trailing only breast cancer in women and lung cancer in men 54. In response to the current dilemma of conventional treatments, scientists have attempted to develop effective therapeutic alternatives. They discovered that ferroptosis is essential for preventing CRC progression and, thus, can be a target for future anticancer treatment 55.

Increased iron concentration is a crucial characteristic of cells that may undergo ferroptosis, because in accordance with the three elements of ferroptosis, iron is an accomplice in the occurrence of ferroptosis 56. Iron is strongly associated with the development of several tumors, the most significant of which being CRC 57. Studies have shown that while iron-deficient CRC patients have a worse prognosis and a lower response to treatment, excess gut luminal iron contributes to the development and progression of CRC 58. Elevated or depleted levels of unstable intracellular iron induce complete growth arrest and segregation of different CRC cell types 59. Therefore, balancing optimal iron intake to avoid iron deficiency and iron overload may be a way to improve the prognosis of patients with CRC 60. Currently, the main forms of iron supplementation include intravenous iron and oral iron, with intravenous iron achieving better clinical outcomes 61. Besides, increasing the level of ROS, the perpetrator of ferroptosis, in CRC cells to induce cancer cell death is also a promising approach to inhibit CRC progression 62. For example, activation of p38 mitogen-activated protein kinase (MAPK) by cetuximab inhibits a major regulator of antioxidant transcription factors, nuclear factor erythroid 2-related factor 2 (Nrf2), thereby increasing Ras-selective lethal small molecule 3 (RSL3)-induced lipid ROS, leading to ferroptosis in CRC cells 62. This finding is expected to contribute to the development of attractive therapeutic strategies for patients with KRAS-mutated CRC: cetuximab combined with ferroptosis inducers 62. However, it is worth noting that a moderate amount of ROS contributes to tumorigenesis and progression by regulating numerous signaling pathways, and it is an excessive amount of ROS can cause ferroptosis and other forms of programmed cell death 63. In summary, it is necessary to monitor specific iron intake or intracellular ROS levels when utilizing iron or ROS to treat CRC, in order to prevent counterproductive effects.

In addition, targeting the ferroptosis defense signaling pathway is a novel strategy for CRC treatment. GSH, as an important component in the GPX4-GSH signaling pathway, is observed the chemotherapy resistance resulting from its elevated levels in human CRC cell lines 64. GSH shortage is a key characteristic of ferroptosis, and cancer cells may be more sensitive to the effects of anticancer drugs if the GSH antioxidant defense system is impaired 65. Therefore, blocking the ferroptosis defense pathway becomes an important strategy to inhibit CRC. It was found that the use of sodium butyrate on CRC cell lines decreased intracellular GSH concentration and caused apoptosis in CRC cells 66. GPX4 in the GPX4-GSH signaling pathway is another important factor in the modulation of ferroptosis 67. Since the cofactors of GPX4 are not restricted to GSH, direct targeting GPX4 may be more effective than GSH-disrupting therapy 68. Application of mollugin, a phytochemical isolated from Rubia cordifolia L., in CRC cell lines reduced GPX4 to inhibit CRC cell proliferation and displayed favorable anticancer outcomes 69. The ferroptosis inducer RSL3 drives ferroptosis by inactivating GPX4 in CRC, leading to CRC cell death 70. These results provide further evidence that GPX4 expression reduction and ferroptosis induction can both effectively hinder the progression of CRC. Targeting other signaling pathways, such as the FSP-CoQ, DHODH-CoQ, and GCH1-BH4 signaling pathways, in addition to the GPX4-GSH system, may also be effective for CRC treatment 34, 71. For example, the combination of the GCH1 inhibitor and the ferroptosis inducer erastin can synergistically inhibit CRC growth *in vivo* 34. When considered collectively, targeting the ferroptosis defense system may affect the growth of CRC, offering novel therapeutic options for CRC management. Some of the studies targeting the ferroptosis defense signaling pathway to inhibit CRC progression are listed in **Table 1**. However, current therapeutic strategies targeting the ferroptosis defense signaling pathway mainly focus on the GPX4-GSH signaling pathway, with the other signaling pathways rarely engaged. Moreover, many drug targets are not limited to molecules in the ferroptosis defense signaling pathway, and the inhibition of CRC progression is the result of the joint action of multiple pathways, implying that we should focus on the combination of multiple targets in order to achieve better therapeutic outcomes.

Ferroptosis can also influence CRC progression and therapeutic efficacy by orchestrating tumor immunity 72. Activated CD8^+^ T cells secrete high levels of interferon-γ (IFN-γ) to induce ferroptosis in tumor cells 73. However, IFN-γ-mediated ferroptosis is triggered at low levels in tumor cells due to limited IFN-γ secretion by CD8^+^ T cells in the immunosuppressive tumor microenvironment 73. Research has indicated that ferroptosis contributes to immune-supportive responses in CRC, with IFN-γ playing a crucial role 72. Therefore, targeting IFN-γ may be another potential therapeutic approach to trigger ferroptosis in CRC cells and thus improve the prognosis of CRC patients, but extensive experiments are still needed to validate this. Of note, the liver, an immune organ, is one of the most common organs for CRC metastasis and colonization, so the role of ferroptosis in the hepatic immune microenvironment may provide novel strategies for the treatment of CRC liver metastasis (CRLM) 74-76. In liver metastases, ferroptosis induces activation and infiltration of CD8 T cells, triggering a tumor-suppressive CD8^+^ T cell response 77. However, ferroptosis can similarly induce immunosuppression by stimulating myeloid-derived suppressor cells (MDSCs) recruitment, thus suggesting that combining ferroptosis induction with MDSCs blockade could be a promising therapeutic tool for CRLM 77. Subsequent *in vivo* experiments confirmed the effectiveness of this combination therapy for CRLM 77. The preceding experimental results illustrate the complex interaction between ferroptosis and tumor immunity and provide a theoretical framework for targeting ferroptosis to treat CRC primary tumors or metastases.

Taken together, different molecules in ferroptosis can be used as potential therapeutic targets for CRC treatment, providing more possibilities for current therapeutic approaches. However, further research is needed on whether targeting these molecules to induce ferroptosis in CRC cells will also damage normal cells or tissues and how to minimize the damage.

## Combination of targeting ferroptosis with other non-operative therapies for CRC treatment

In addition to the potential of targeting ferroptosis alone in CRC treatment, combining targeting ferroptosis with non-operative therapies, including chemotherapy, radiotherapy, and immunotherapy, can improve the effectiveness of non-operative therapies, opening the door to a new world for comprehensive CRC management 82.

Oxaliplatin is a widely used chemotherapeutic agent in patients with advanced CRC, but frequent resistance limits its therapeutic efficacy 83. Cyclin-dependent kinase 1 (CDK1) was found to be a key factor in oxaliplatin resistance because CDK1 mediates the degradation of Acyl-CoA synthetase long-chain family 4 (ACSL4) to block the process of ACSL4, inhibiting lipid peroxidation and ferroptosis, which leads to drug resistance 84. Furthermore, *in vivo* and *in vitro* experiments demonstrated that treatment with ferroptosis inhibitors reduced the enhanced sensitivity of CRC cells to oxaliplatin, which is achieved through CDK1 inhibition 84. This suggests that the combination of ferroptosis inducers with chemotherapy may be another means of CRC treatment.

Moreover, radiotherapy is also often limited by the occurrence of radioresistance 85. The overexpression of long non-coding RNA ovarian tumor domain-containing 6B-antisense RNA1 (lncRNA OTUD6B-AS1) stabilizes the tripartite motif 16 (TRIM16) mRNA by binding to the RNA-binding protein human antigen R and increases TRIM16 mRNA levels, promoting GPX4-mediated ferroptosis and inhibiting radioresistance in CRC cells; however, inhibiting ferroptosis attenuates the inhibitory effect of overexpressed lncRNA OTUD6B-AS1 in CRC radioresistance 86. This finding promises to eliminate the limitations of CRC radiotherapy and enhance the therapeutic effect.

In 2017, immune checkpoint therapy received regulatory approval for the treatment of a small fraction of patients with CRC (microsatellite instability high or mismatch repair deficient) 87. For most other types of CRC, the immune checkpoint inhibitors (ICIs), programmed cell death 1 (PD1)-, or programmed cell death 1 ligand 1 (PDL1)-blocking antibodies, are ineffective 87. Apolipoprotein L3 (APOL3) overexpression can enhance RSL3-induced ferroptosis and improve the therapeutic efficacy of PDL1 inhibitors 88. Additionally, CYP1B1 overexpression can promote ACSL4 ubiquitination and degradation, making CRC cells resistant to ferroptosis and anti-PD1 therapy, whereas CYP1B1 inhibition promotes ferroptosis, making CRC cells susceptible to anti-PD1 antibodies 51. These studies may broaden the application of ICIs in CRC treatment.

In summary, combining ferroptosis and other non-operative therapies can reverse drug resistance or broaden the scope of application to compensate for the limitations of the existing approaches, providing a novel concept for comprehensive CRC treatment.

## Role of ferroptosis-related biomarkers or genes in CRC early diagnosis and prognosis

Although surgical and pharmacological treatments have improved the prognosis of CRC patients, the 5-year survival rate of patients is still unsatisfactory 89. The time of the diagnosis and the stage at which the disease is discovered are critical factors in determining CRC prognosis: Stage I has a 5-year survival rate of up to 90%, whereas that for stage IV is less than 10% 7. Accordingly, it is important to improve the effectiveness of early diagnosis.

Nowadays, imaging and stool-based tests are the primary techniques of CRC screening, including colonoscopy, stool-based tests, Cologuard (a stool DNA test), flexible sigmoidoscopy, and computed tomographic colonography 7. Due to variations in sensitivity, specificity, cost, time, and patient tolerance, different tests cannot be used routinely in all patients 90. Therefore, further studies should be conducted to investigate novel biomarkers that can be applied in the early detection and diagnosis of CRC.

Given the role of ferroptosis in the progression and treatment of CRC, its potential in the early diagnosis and prognosis of CRC is being progressively explored 14, 91. Many studies have provided evidence that ferroptosis-related biomarkers may be useful in the early diagnosis of CRC 14. The transferrin dipstick appears to be a highly sensitive test for detecting not just cancer but also precancerous lesions, providing an additional tool for CRC screening with an overall accuracy of 76.4% for detecting CRC and precancerous lesions 14. Ferritin, a major intracellular iron storage protein complex, is another protein linked to ferroptosis and may serve as a biomarker for CRC diagnosis 92, 93. However, its diagnostic specificity is low and needs to be used in combination with other serum markers to diagnose early CRC 93. These molecules may have potential in the early diagnosis of CRC, and additional research is required to confirm this possibility and improve their diagnostic specificity and sensitivity.

Many recent studies have focused on the development of prognostic profiles of ferroptosis-related genes (FRGs) to predict the prognosis and treatment response of CRC patients 91, 94. For instance, Shao et al. established the 10 FRGs signature (TFAP2C, SLC39A8, NOS2, HAMP, GDF15, FDFT1, CDKN2A, ALOX12, AKR1C1, and ATP6V1G2) that may accurately predict the prognosis and survival time of CRC patients 95. **Table 2** summarizes the role of FRGs signature in CRC prognosis. These models may offer useful information to predict the prognosis of CRC patients, although the roles of individual FRG in these models are not fully understood. In addition, the aberrant expression of a single FRG may affect the disease prognosis 76, 96. The expression of metallothionein-1G (MG1T) reduced significantly in CRC tissues 96. Patients with CRC with high MT1G levels had a worse prognosis and aberrantly expressed MT1G affected the immune response 96. Consequently, FRGs have the potential to predict the prognosis of patients with CRC.

In summary, the discovery of ferroptosis-related biomarkers and genes may improve the prognosis and survival of patients with CRC. Future research should focus on establishing an ideal prognostic model and identifying key biomarkers, which will be crucial for accurately predicting CRC prognosis and early diagnosis.

## Conclusion

Ferroptosis, a newly discovered form of cell death, has received widespread attention from the scientific community and is becoming a hot topic in oncology and anticancer therapeutic research. The link between the occurrence and treatment of CRC, a common type of malignant tumor, and ferroptosis is being widely explored. Scientists have investigated the role of ferroptosis in CRC progression using its three elements and defense signaling pathways. Moreover, a number of drugs targeting ferroptosis have been found to treat CRC and improve patient outcomes. Molecules associated with ferroptosis have also been explored for their potential in treating CRC and are likely to serve as therapeutic targets. The discovery of multiple biomarkers and the development of predictive models have also aided in the early diagnosis and prognosis of patients with CRC. However, some unresolved questions remain about the regulatory mechanism of ferroptosis and its applicability in CRC: (1) Ferroptosis susceptibility factors exist as a double-edged sword in CRC cells, and their up-regulation may cause ferroptosis while simultaneously providing CRC cells with extra raw materials needed for proliferation. Therefore, it is insufficient to target these factors; instead, we should target the intermediate molecules that cause these two different outcomes, necessitating a more in-depth examination of the mechanisms involved. (2) When targeting ferroptosis for CRC treatment, the uncertainty of the effective targets of ferroptosis should be resolved by choosing the appropriate targets among multiple potential ferroptosis-related molecules and clarifying the appropriate drug dosage. (3) The acceleration of translating basic ferroptosis research findings into clinical applications is crucial for enabling early diagnosis, individualized treatment, and precise prognosis.

## Figures and Tables

**Table 1:** Selected studies targeting ferroptosis defense signaling pathways to inhibit CRC progression in the last five years.

Drug	Target	Mechanism and result	Research type	References
NaB	GSH	NaB can reduce intracellular GSH concentration to induce apoptosis in CRC cells.	Cell experiment	66
GRh3	GSH	GRh3 leads to the depletion of GSH and thus induces ferroptosis in CRC cells, effectively inhibiting the proliferation of CRC cells.	Cell and animal experiment	78
Mollugin	GPX4	Mollugin can reduce GPX4 to inhibit the proliferation of CRC cells, showing favorable anticancer effects.	Cell experiment	69
curcumin and andrographis	GPX4	Curcumin and andrographis combination therapy exhibited anticancer effects in CRC cells by activating ferroptosis via inhibiting GPX4.	Cell experiment	79
RSV	GPX4	RSV can promote ferroptosis and effectively inhibit the growth of CRC cells by down-regulating the expression of GPX4.	Cell and animal experiment	80
β-elemene and cetuximab	GSH and GPX4	The combination of β-elemene, a natural product isolated from Chinese herbs, and cetuximab induces ferroptosis by targeting ferroptosis-associated molecules, such as GSH and GPX4, to inhibit KRAS-mutant CRC growth and metastasis.	Cell and animal experiment	81
leflunomide	DHODH	Leflunomide inhibits DHODH and DHODH depletion significantly reduces CRC liver metastasis colonization.	Animal experiment	71
DAHP	GCH1	Inhibition of the GCH1/BH4 signaling pathway by DAHP, a specific inhibitor of GCH1, promoted erastin-induced ferroptosis, suggesting that the combination of a GCH1 inhibitor and erastin is a novel therapeutic strategy for the treatment of CRC.	Cell and animal experiment	34

**Abbreviations:** DAHP, 2,4-diamina-6-hydroxypyrimidine; GRh3, Ginsenoside Rh3; KRAS, Kirsten rat sarcoma virus; NaB, sodium butyrate; RSV, resveratrol.

**Table 2:** Role of FRGs signature in CRC prognosis.

Model	FRGs	Role in the prognosis of CRC	References
3-gene prognostic model	CDKN2A, FDFT1, and ACSL6	Predict prognosis of CRC and assess immune response	97
3-gene prognostic model	ACACA, GSS, and NFS1	Improve individual prognostic monitoring and provide new ferroptosis-related treatment strategies for CRC patients	98
3-gene prognostic model	ATG7, MAPK9, and MMD	Predict immunotherapy responses and help determine CRC treatment strategies	99
4-gene prognostic model based on EMT and FRGs	MMP7, YAP1, PCOLCE, and HOXC11	Recognize metastatic COAD	100
10-gene prognostic model	TFAP2C, SLC39A8, NOS2, HAMP, GDF15, FDFT1, CDKN2A, ALOX12, AKR1C1, and ATP6V1G2	Effectively predict the prognosis and survival time of CRC patients, and provide clinical therapeutic benefits for targeted therapy or immunotherapy	95
10-gene prognostic model	ATG7, DUOX1, NOX4, PGD, TP63, ATP6V1G2, DRD4, JDP2, SLC2A3, and VEGFA	Serve as an individualized and more accurate survival prediction tool for CRC patients	101

**Abbreviations:** COAD, colon adenocarcinoma; EMT, epithelial-mesenchymal transition.

## Abbreviations

ACSL4: Acyl-CoA synthetase long-chain family 4
AKT: protein kinase B
APOL3: apolipoprotein L3
BH4: tetrahydrobiopterin
CDK1: cyclin-dependent kinase 1
CoQ: coenzyme Q
CRC: colorectal cancer
CRLM: CRC liver metastasis
CYGB: cytoglobin
CYP1B1: cytochrome P450 1B1
DHODH: dihydroorotate dehydrogenase
FRGs: ferroptosis-related genes
FSP1: ferroptosis suppressor protein 1
GCH1: guanosine 5'-triphosphate cyclohydrolase-1
GPX4: glutathione peroxidase 4
GSH: antioxidant glutathione
ICIs: immune checkpoint inhibitors
IFN-γ: interferon-γ
lncRNA OTUD6B-AS1: long non-coding RNA ovarian tumor domain containing 6B-antisense RNA1
LOX: lipoxygenase
MAPK: mitogen-activated protein kinase
MDA: malondialdehyde
MDSCs: myeloid-derived suppressor cells
MG1T: metallothionein-1G
Nrf2: nuclear factor erythroid 2-related factor 2
PD1: programmed cell death 1
PDL1: programmed cell death 1 ligand 1
PI3K: phosphatidylinositol 3-kinase
PTEN: phosphatase and tensin homolog deleted on chromosome 10
PUFAs: polyunsaturated fatty acids
RCD: regulated cell death
ROS: reactive oxygen species
RSL3: Ras-selective lethal small molecule 3
TfR1: Transferrin receptor 1
TIGAR: TP53-induced glycolysis and apoptosis regulator
TRIM16: tripartite motif 16
